# Adulthood Employment Trajectories and Later Life Mental Health before and after the Onset of the COVID-19 Pandemic

**DOI:** 10.3390/ijerph192113936

**Published:** 2022-10-26

**Authors:** Ignacio Cabib, Carlos Budnevich-Portales, Ariel Azar

**Affiliations:** 1 Instituto de Sociología, Pontificia Universidad Católica de Chile, Santiago 7820436, Chile; 2 Departamento de Salud Pública, Pontificia Universidad Católica de Chile, Santiago 7820436, Chile; 3 Centro UC Estudios de Vejez y Envejecimiento, Santiago 7820436, Chile; 4 Departamento de Economía, Facultad de Economía y Negocios, Universidad de Chile, Santiago 8330015, Chile; 5 Department of Sociology, University of Chicago, Chicago, IL 60637, USA

**Keywords:** employment trajectory, longitudinal analysis, later life mental health, COVID-19, Chile

## Abstract

Background: This life course study has two aims. First, to explore how diverse employment trajectories across adulthood are related to older people’s mental health in Chile, a country with no research in this field, and second, to analyze these associations before and after the onset of the COVID-19 pandemic. Methods: We use data from the nationally-representative and longitudinal ‘Chilean Social Protection Survey’ sequence analysis to reconstruct employment trajectory types, and bivariate and multivariate analyses to measure their association with depressive symptoms. Results: Our findings indicate that formal labor force patterns in adulthood show the lowest burden of depressive symptomology before and after the onset of the overwhelming COVID-19 pandemic when controlling for traditional risk factors. Conclusion: We emphasize that policymakers in both the labor market and public health domains must consider the relationship between informal employment pathways in adulthood and poorer mental health in old age. Public policies should improve the conditions and quality of jobs during adulthood and promote more formalization in the labor market to address the high uncertainty involving low social protection, which is strongly associated with severe mental health problems in later life.

## 1. Introduction

Besides their increased mortality risk, one of the main consequences of the COVID-19 pandemic among older people involves their mental health and well-being [1]. Recent studies have emphasized that, as occurred before the onset of the pandemic [2], social factors such as gender, marital status, and social contact frequency strongly affected mental health status in older adults during the pandemic. Specifically, women [3], unmarried people [4], and those with low peer interaction [5,6] have shown more psychological distress and depressive symptomatology in later life.

Scholars have also shown that employment status has strongly influenced mental health problems among older people during this period. Factors such as involuntary job loss, reduction of individual and household income, and feelings of economic uncertainty have led older adults to face symptoms of anxiety [7], depressive symptoms [8,9], feelings of loneliness [10,11], and stress [12,13]. In addition, compared with full-time workers, older people who were retired, unemployed, or in a part-time job immediately before the onset of the pandemic are more likely to have worsened their mental health [14,15].

### 1.1. Research Gaps

While these studies provide rich evidence on later-life employment and mental health problems, their cross-sectional design is a relevant limitation. This is problematic for two reasons. First, before the pandemic, most cross-sectional evidence on the association between employment and mental health has proven to be equivocal or ambivalent. On the one hand, some studies indicate that older people engaged in paid work show better mental health indicators [16,17], while unemployed people are more likely to feel loneliness and depression [18] due to the health-protective effect of material and non-material characteristics of jobs [19]. On the other hand, research has indicated that working in later life could have detrimental effects on mental health [20,21,22] or even that there is no association between employment status and mental health among older individuals [23].

Second, by exploring both employment and mental health at a specific time point during the COVID-19 pandemic, cross-sectional studies neglect the effect of having been exposed to diverse employment trajectories across the life course, with either longer or shorter spans within the labor market, in full-time or part-time jobs, or in formal or informal employment, as defined by the (in)existence of a formal work contract or contributions to social security based on employment status [24,25]. Therefore, these studies prevent us from understanding how the adverse exogenous impact of the pandemic has had different effects on the mental health of older individuals with diverse employment pathways. As Settersten and other remarkable life course scholars have stressed [26], p. 10: “*the pandemic brings to the fore how individuals have different susceptibility to the virus itself and to the social and economic consequences of the pandemic, depending on their previous experiences—experiences that can also determine the short-term and long-term consequences of the pandemic*”.

### 1.2. The Study Field of Employment Trajectories and Later Life Health

Over the past 15 years, based on a life course approach [25,27], an increasing number of longitudinal studies have addressed the association between long-term employment trajectories and subsequent health in old age. A recent paper summarized the main findings of 17 studies on this matter [28]. Across different populations, older individuals who followed advantaged employment pathways (composed of stable and formal full-time jobs along with sufficient income) show a better sense of control, improved autonomy and self-realization, greater pleasure, better self-rated health, higher subjective well-being, fewer functional limitations, better oral health, lower mortality risk before the age of 75, lower markers of stress and inflammation, better metabolic markers, and lower levels of frailty after the age of 60 [29].

Regarding mental health indicators, longitudinal studies have provided more consistent results than cross-sectional research. Specifically, longitudinal studies show that advantaged employment pathways are systematically associated with better mental health in old age, including fewer mental illnesses and depressive symptoms [5,24,30,31,32]. Put in other words, constantly being out of the labor market across adulthood [33] or permanently working under stressful conditions [34] or adverse conditions (i.e., involuntary redundancy, weak labor market ties, and disadvantaged occupational status [27,35] lead to poor mental health in late life. According to these authors, the subsequent positive health outcomes among those following advantaged employment pathways are due to persistent adequate labor conditions that allow people to access better financial status, medical care, health services, and housing conditions, as well as to experience lower stress levels and higher community integration across their lives.

However, most notable research on employment trajectories and mental health in later life has been conducted predominantly in developed Western countries [27,35]. Most older people in these settings benefited across their adulthood from post-war financially stable economies and pension systems, large formal labor markets, and robust health policies. By contrast, during their lives, many older individuals in developing countries such as Chile in the 20th century have faced inadequate welfare state provisions, economic and pension systems with intense fiscal pressures, weak public healthcare services, and, importantly, large informal sectors in the labor market that prevent individuals from having access to benefits such as unemployment insurance, better social health insurance, and higher pensions in old age [36]. Therefore, it is also crucial for this field of research to explore the association between working patterns and prospective mental health in these developing settings to understand whether older people in these countries follow different trends to those in developed nations.

### 1.3. Current Study

Drawing on a life course perspective and relying on rich longitudinal quantitative data that allowed us to “*link information on life before COVID-19 to experiences during and specific to the pandemic*” (as encouraged by Settersten et al. [26], p. 9), this study has two research aims. First, to explore how diverse employment trajectories across adulthood (ages 30 to 60) are either positively or negatively related to older people’s mental health in Chile, including a well-known scale of depressive symptoms. Second, to analyze these associations before and after the onset of the pandemic. In other words, to analyze whether older people’s mental health reacted differently to the adverse contextual circumstances of COVID-19 depending on their employment trajectories in adulthood, as the relationship between their previous life experiences and mental health could have been exacerbated by the pandemic.

## 2. Materials and Methods

### 2.1. Longitudinal Data

To address the two aims of this study, we use data from a rich and nationally-representative longitudinal survey called the ‘Chilean Social Protection Survey’ (*Encuesta de Protección Social*, EPS, in Spanish). EPS is the oldest and largest face-to-face panel survey (N = ~14,000 individuals aged 18+), and it is focused on multiple aspects of the life course such as educational transitions, marital transitions, employment trajectories, health problems, childhood socioeconomic adversities, and subsidies and public transfers, among others.

So far, within the framework of this panel survey, six valid waves have been conducted, in 2002, 2004, 2006, 2009, 2015, and 2020. Because of the COVID-19 pandemic, the 2020 wave was conducted in two stages: first, through face-to-face surveys before the pandemic between December 2019 and March 2020, and second, through telephone surveys during the pandemic between August and December 2020.

### 2.2. Survey Waves Used in This Study and Sample Derivation

On the one hand, to reconstruct the employment trajectories, we used the EPS waves for 2002, 2004, 2006, 2009, and 2015. Each of these waves included a module on retrospective employment histories. These modules provide information on labor force participation, employment status, and periods of unemployment and inactivity. Individuals were asked about their work histories from 1980, starting at age 15, until the date of the first wave of each respondent. Then, during the following waves, individuals were requested to provide labor force information between the period between waves, for example, from 2002 to 2004, then from 2004 to 2006, then from 2009, and then from 2009 to 2015. During the face-to-face interview, the interviewer recorded the start date (year and month) of each period (whether employment, unemployment, or inactivity) and, if applicable, the end date (year and month) of each period. The data collection involved the use of life history calendars that helped respondents to remember and chronologically organize the various episodes during their working lives.

The analysis of employment trajectories was conducted in a previous study (see [28]) that resulted in the identification of a representative typology of seven adulthood employment trajectories (from age 30 to 60). For this purpose, we used sequence analysis. Two criteria were used to select these individuals: they had to be aged 30 years old at the beginning of the observation period (between 1980–1985) and they had to have 30 years of employment history information (until 2010–2015) in the life history calendars. Among the ~14,000 individuals aged 18+ originally sampled in 2002, 3782 individuals met these two selection criteria.

The description of the typology of employment trajectories is explained in the Variables subsection. For simplicity, the data containing the employment trajectories will henceforth be called ‘baseline data’.

**Figure 1:**
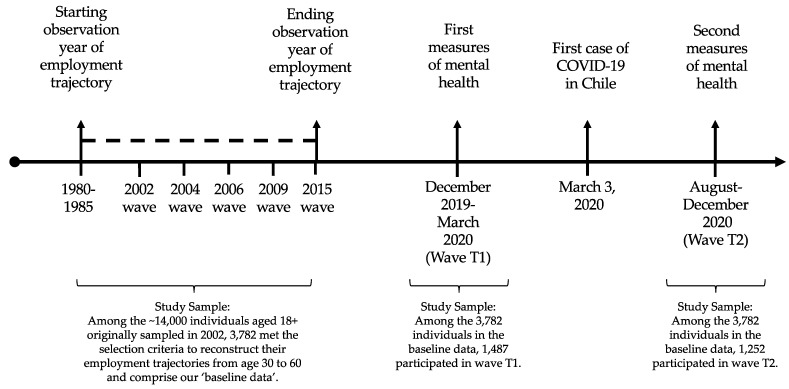
Survey waves used in this study and sample derivation.

On the other hand, we measured mental health indicators at two time points; once before the onset of the COVID-19 pandemic, in the first stage of the 2020 wave (henceforth ‘wave T1′), and then once after the onset of the pandemic, in the second stage of the 2020 wave (henceforth ‘wave T2′). Figure 1 illustrates the survey waves used, the sample derivation, and the time frame of the study.

### 2.3. Study Samples

An important aspect to define the study sample is that, as commonly occurs in panel surveys subject to attrition, of the 3782 individuals belonging to the baseline data, only 457 were observed both in waves T1 and T2. In this relatively small sample, certain sociodemographic groups and types of employment trajectories are overrepresented. This leads to sample selectivity issues and therefore makes it difficult to perform a longitudinal analysis based on panel methods.

Instead, we propose to carry out a longitudinal analysis, but based on cross-sectional methods, that is, to merge the baseline data of employment trajectories separately with waves T1 and T2. The sample sizes after merging the baseline data with waves T1 and T2 resulted in 1487 and 1252 older individuals, respectively. In order to be sure that we are not dealing with sample selectivity issues, in the description of the employment trajectory typology in the Variables subsection and of the univariate statistics in the Results section, we show how the proportion of the key variables of this study remain highly similar across the merged samples.

### 2.4. Measures

#### 2.4.1. Independent Variable: Employment Trajectories in Adulthood

The typology of seven adulthood employment trajectories identified [28] was constructed based on four labor force indicators: (i) employment condition (whether individuals worked or not, including inactivity, unemployment, and periods of job search); (ii) time-based employment (whether they worked full-time (more than 30 h per week) or part-time (30 h or less per week)); (iii) employment status (whether they worked as dependent employees or were self-employed); and (iv) formality status, measured as whether people contributed to social security or not. This is a key indicator of job formality in Chile, a context where the contributory pillar of the pension system is based exclusively on individual retirement accounts. Those who are formally employed contribute to social security through their employers and are subject to all labor regulations. Those who are dependent employees but do not have a formal contract do not contribute to social security and are not protected by any labor regulations. Regarding the self-employed, because contributions to social security were voluntary during this cohort’s working life, those who contribute have access to social protection in the form of health insurance and retirement saving accounts, while those who do not only have access to basic health insurance and a basic pension in old age [28]. In Table 1 we show the title, the description, and the proportion in the baseline and merged samples of each of the seven types of employment trajectories.

#### 2.4.2. Dependent Variables: Depressive Symptoms before and after the Onset of the Pandemic

In waves T1 and T2 we evaluated the nine-item Patient Health Questionnaire (or PHQ-9). This is a test, validated in Chile [37], used to measure and identify depressive symptomatology based on common mental disorders such as feeling down, depressed, hopeless, having trouble falling or staying asleep, sleeping too much, feeling bad about oneself, or having thoughts that one would be better off dead [38]. Each of these items is answered using the following Likert four-response scale: ‘not at all’ (value 0), ‘several days’ (value 1), ‘more than half the days’ (value 2), and ‘nearly every day’ (value 3). A summative score ranging from 0 to 27 thus comprises the continuous PHQ-9 scale. We use this continuous scale, as well as two categorical versions of it, mainly to have a more informative report of the severity of depressive symptoms [38]. First, a five-level depression severity scale: ‘none’ (score 0 to 4), ‘mild’ (score 5 to 9), ‘moderate’ (score 10 to 14), ‘moderately severe’ (score 15 to 19), and ‘severe’ (score 20 to 27); and second, a two-level depression severity scale: ‘non-depressed’ (score 0 to 9), and ‘depressed’ (score 10 to 27).

#### 2.4.3. Control Variables

Following a conservative strategy, our analyses are adjusted by multiple variables that are typically related to mental health: marital status (‘divorced/separated’, ‘married/partnered’, ‘single’, ‘widowed’), number of children, education level (‘none or primary‘, ‘secondary’, and ‘tertiary’), household income decile, age, gender (‘male’, ‘female’), tobacco use (‘yes’, ‘no’), beer consumption (‘yes’, ‘no’), wine consumption (‘yes’, ‘no’), liquor consumption (‘yes’, ‘no’), number of functional limitations (0 to 7, including difficulty to perform strenuous or intense exercise, walking long distances, climbing stairs, bathing, dressing, eating, and getting out of bed), and number of chronic conditions (0 to 8, including high blood pressure or hypertension, diabetes or high blood sugar, cancer or malignant tumor, chronic lung disease, heart problems, arthritis or rheumatism, kidney disease, and stroke).

### 2.5. Statistical Analysis

First, we estimated the bivariate relationship between adulthood employment trajectories and the mental health outcomes in waves T1 and T2, using bivariate association techniques along with the visual support of several figures. Then, in order to understand whether employment trajectory types are significantly related to mental health, even under the influence of control variables, we estimated cross-sectional multivariate linear regression models at the two time points of interest. In these models, we focus only on continuous measures of dependent variables. We also measured the multicollinearity among all our predictors and none of them showed a variance inflation factor (VIF) higher than 2. All our analyses were conducted in the statistical software R [39].

## 3. Results

### 3.1. Univariate Descriptive Statistics

Table 2 shows descriptive statistics of mental health outcomes and genders across the baseline waves T1 and T2, and merged samples of employment trajectories with the T1 and T2 waves (see Appendix A for descriptive statistics of control variables for the whole sample and for each employment trajectory type). In Table 2, we distinguish the wave collected before the onset of the pandemic (T1) from the wave collected after it (T2). It can be observed that there are no sample selectivity issues, as these key variables remain highly similar across samples (as occurred with the employment trajectories in Table 1).

In merged sample T1, the mean score of the continuous PHQ-9 scale is 4.5. The five-level PHQ-9 scale indicates that 63.1% had no depressive symptoms, while the remaining 36.9% presented either mild, moderate, moderately severe, or severe depression levels. When using the more conservative two-level PHQ-9 scale, 84.7% of older persons are classified as non-depressed, while 15.3% are classified as depressed.

In merged sample T2, the mean score of the continuous PHQ-9 scale significantly rose to 6.1. Consequently, the five-level PHQ-9 scale indicates that only 51.3% of older persons presented no depressive symptoms, while 48.7% were classified in one of the four levels of depression. Accordingly, the two-level PHQ-9 scale shows that 25.5% were characterized as depressed.

Finally, the proportion of women across all merged sample ranges is approximately 57%, while that of men is approximately 43%.

### 3.2. Bivariate Associations between Employment Trajectories and Mental Health Outcomes

**Figure 2:**
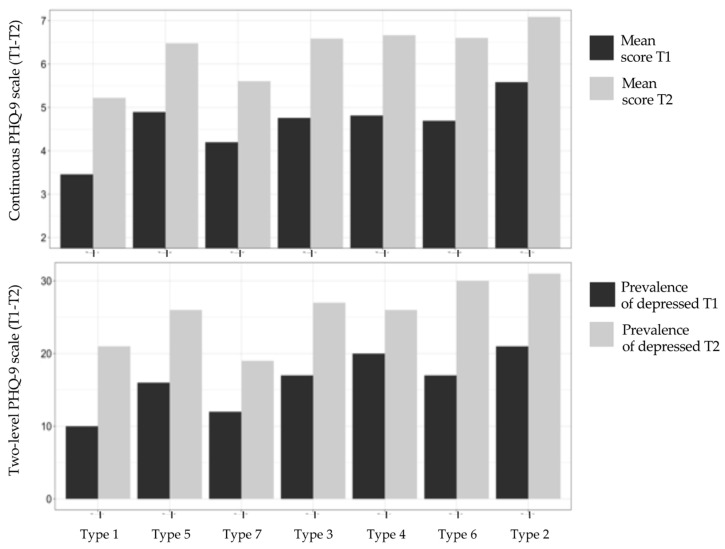
Mental health outcomes by employment trajectory type before and after the onset of the COVID-19 pandemic. Note: Formal employment trajectories: Type 1, Type 5, and Type 7. Informal employment trajectories: Type 3, Type 4, and Type 6. Non-employment trajectory: Type 2.

Figure 2 and Appendix B show the bivariate association between the seven types of employment trajectories and the mental health outcomes before the onset of the pandemic in wave T1, and after the onset of the pandemic in wave T2. For the sake of simplicity, in the table in Appendix B and Figure 2, we sort the results of employment trajectories based on their type of labor force attachment: *formal employment trajectories*, which share a high prevalence of formal work (*Type 1, Conventional work life cycle; Type 5, Full-time self-employed contributing;* and *Type 7, Part-time wage-earners contributing*); *informal employment trajectories,* which feature informal work patterns with no pension contributions (*Type 3, Full-time self-employed not contributing; Type 4, Wage-earners not contributing; and Type 6, Part-time self-employed not contributing*); and *non-employment trajectories*, which have extended periods out of the labor force (*Type 2, Out of the labor force*). We assessed the bivariate relationship between employment trajectory types and dependent variables using ANOVA and chi-square tests, and a Poisson regression to estimate prevalence ratios. It should be noted that only in multivariate analyses (conducted below) do we adjust our models by traditional risk factors of mental health.

#### 3.2.1. Formal Employment Trajectories

Overall, Figure 2 indicates that trajectories of formal employment types show the lowest prevalence of depressive symptoms measured using the continuous and categorical scales. Specifically, all trajectory types show scores on the continuous PHQ-9 scale that increased between 1.4 and 1.7 points before and after the onset of the pandemic. However, trajectory type 1 (full-time formal dependent employees) shows the lowest scores at both time points, with 3.5 and 5.2 points, followed by trajectory Type 7 (part-time formal dependent employees), with scores of 4.2 and 6.5, and then trajectory Type 5 (full-time formal self-employed), with scores of 4.9 and 6.5.

#### 3.2.2. Informal Employment Trajectories

The trajectory types included in this second group show worse mental health scores than formal employment trajectories. Specifically, the increase on the continuous PHQ-9 scale before and after the onset of the pandemic is slightly higher than in formal employment trajectories, with a change of between 1.8 and 1.9 points. In terms of the actual scores on this continuous scale, the trajectory types changed from 4.7–4.8 to 6.6–6.7. Then, using the more conservative two-level PHQ-9 scale, trajectory Type 3 (full-time informal self-employed) shows an increase of 9.7 percentage points in the proportion of depressed individuals (from 17.4% to 27.1%), trajectory Type 4 (informal dependent employees) displays an increase of 6.4 percentage points (from 19.7.% to 26.1%), and Type 6 (part-time informal self-employed) shows an increase of 12.8 percentage points (from 17.4% to 30.2%). At T1, Type 2 shows a significantly higher prevalence of depression relative to Type 1, having a 43% higher probability of reporting depression (see table in Appendix B). In T2, this difference is even bigger.

#### 3.2.3. Non-Employment Trajectories

Trajectory Type 2 (individuals persistently outside the labor market) show the worst mental health indicators, both before and after the onset of the pandemic. It showed an increase of 1.5 points in the score on the continuous PHQ-9 scale before and after the onset of the pandemic, which is smaller than in informal employment trajectories and approximately the same as that in formal employment trajectories. However, this change occurs within a poorer range of mental health scores, from 5.6 to 7.1 points. Accordingly, the proportion of people with either moderately severe or severe depressive symptoms on the five-level PHQ-9 scale grew from 7.2% to 16.4%, and the proportion of those classified as depressed on the two-level PHQ-9 scale rose from 21.4% to 30.8%. Type 4 also shows a significantly higher probability (+83%) of reporting depression relative to Type 1 at T1.

### 3.3. Employment Trajectories and Mental Health Outcomes: Multivariate Regression Analysis

**Figure 3:**
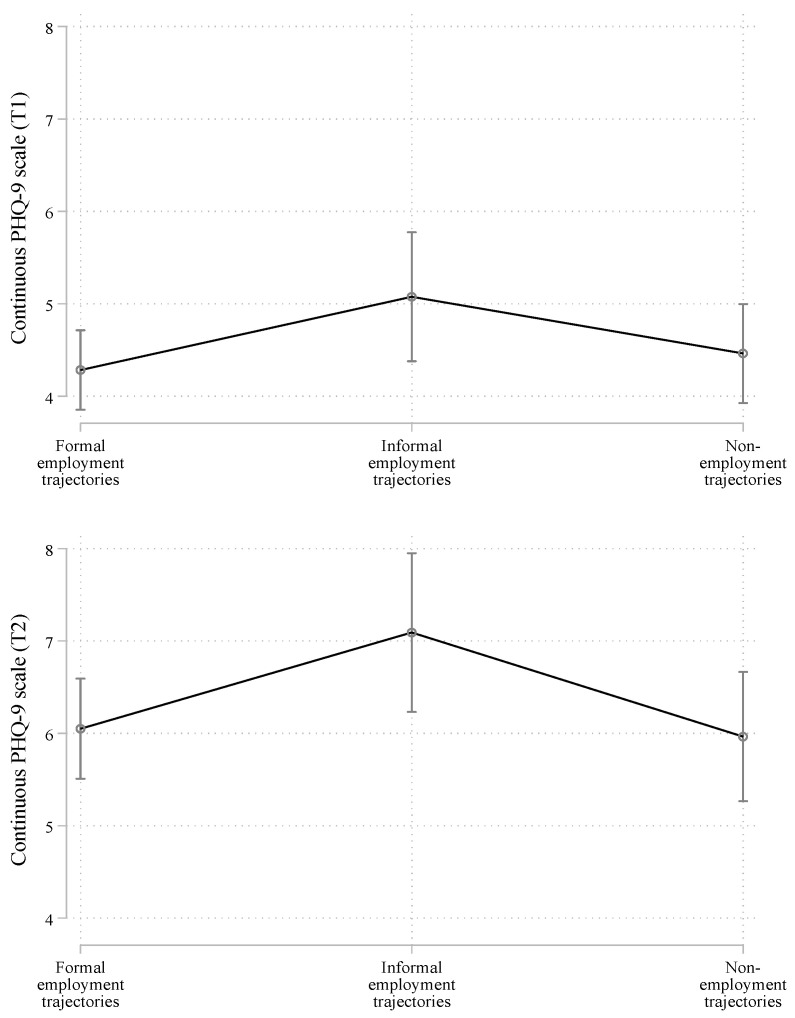
Average marginal effects from multivariate linear regression models on mental health outcomes before and after the onset of the COVID-19 pandemic (collapsed trajectory types). Note: Formal employment trajectories: Type 1 (conventional work life cycle), Type 5 (full-time self-employed contributing), and Type 7 (part-time wage-earners contributing). Informal employment trajectories: Type 3 (full-time self-employed not contributing), Type 4 (wage-earners not contributing), and Type 6 (part-time self-employed not contributing). Non-employment trajectory: Type 2 (out of the labor force). ‘T1′ and ‘T2′ correspond to the result of merging the baseline data with waves T1 and T2, respectively.

Figure 3 and Appendix C show the results of the following linear regression models before and after the onset of the pandemic: one model for the continuous PHQ-9 scale in wave T1 and one model for the continuous PHQ-9 scale in wave T2.

For simplicity, we show the predicted values of the PHQ-9 scale for the adulthood employment trajectories collapsed in the three groups discussed (*formal*, *informal*, and *non-employment trajectories*). However, the regression model for all trajectory types is displayed in Appendix D. In Figure 3 and Appendix C, we show only the results of the employment trajectory types, but the model is adjusted by all the traditional risk factors of mental health mentioned. As a reference value in the independent variable, we use the employment trajectory type showing the most positive association with mental health in the bivariate analysis, that is, *formal employment trajectories*.

Overall, the multivariate regression analysis confirmed that the observed associations in the bivariate analysis were robust, particularly for the *informal employment trajectories*. Specifically, when compared with *formal employment trajectories*, types of informal employment show worse mental health indicators both before and after the onset of the pandemic. As shown, in Appendix D this result is driven by Type *3, full-time self-employed not contributing*. In addition, interestingly, Appendix D indicates that, among the *formal employment trajectories*, trajectory Type 5 (formal full-time self-employed) is more likely than Type 1 (formal full-time dependent employees) to present higher depressive symptoms before the onset of the pandemic.

## 4. Discussion

This study addressed two research aims. The first consisted of exploring how diverse employment trajectories across adulthood (ages 30 to 60) are associated with older people’s mental health in Chile, either positively or negatively.

This is a country where, unlike most developed Western countries, many older people face an economy with strong fiscal pressures, weak public health benefits from the state, and, importantly, high levels of informality in the labor market [36]. Our results indicate that individuals with formal employment trajectories, regardless of whether they work as dependent employees or are self-employed, or work in full-time or part-time jobs, are more likely to show relatively better mental health outcomes.

Thus, permanent formality across the working life (measured in this study as social security contributions) appears to be a key positive factor for the mental health of individuals, even when controlling for multiple traditional risk factors. The stability and social protection involved in these working patterns and the consequent access to better financial status, medical care, health services, and housing conditions [34,35] are plausible mechanisms underlying this association with an advantaged mental health status in old age. In contrast, those who experience persistent informality and erratic working patterns, and those who mostly did not work, tend to replicate their accumulated social and financial disadvantages in worse mental health in later life [33].

The regression models in Appendix D indicate, nevertheless, that being in full-time self-employed formal (Type 5) and informal (Type 3) work is associated with poorer mental health than formal full-time dependent employment. In a context such as that in Chile, where self-employment is particularly precarious, higher pension insecurity due to the absence of adequate policies targeting the self-employed [40] might explain why self-employment is a relatively worse-off position for mental health outcomes when compared with formal dependent employment. Additionally, recurrent lack of experience and lack of risk tolerance to face the challenges of self-employment in old age [41] might also contribute to this relationship.

The second objective of this study consisted of exploring whether older people’s mental health reacted differently to the adverse contextual circumstances of COVID-19, depending on their employment trajectories in adulthood. In short, our results indicate that the rate of increase in mental health problems after the onset of the pandemic was somewhat similar across formal, informal, and non-employment trajectories. In other words, the exogenous impact of the pandemic was similar for most older people’s mental health.

However, a key difference between employment trajectories regarding mental health before and after the onset of the pandemic was the range in which the increase of mental health problems occurred. Specifically, those individuals who faced informality most of their working lives and those who consistently followed trajectories that kept them out of the labor market increased their mental problems into a much higher range than people with formal employment trajectories. This finding reinforces the positive role of working pathways with persistent formality for mental health in old age, which adds to the positive effects on financial outcomes.

We therefore emphasize that policymakers in both the labor market and public health domains must seriously consider the relationship between employment pathways that are either erratic or informal in adulthood and poorer mental health in old age. Specifically, public policies should be aimed at improving the conditions and quality of jobs during adulthood and promote more formalization in the labor market in order to address the high uncertainty involving low social protection, which, as discussed in this study, is related to serious mental health problems in late life.

### Limitations

The results of this study should be considered, taking into account some design limitations. As mentioned, because of the sample attrition in the panel survey used, we opted to conduct a longitudinal analysis at the two time points of interest, but based on cross-sectional methods with different samples. While these samples show similar prevalence in the key variables of the study, they prevent us from being confident that the comparisons represent changes over time. Thus, further research should conduct longitudinal analysis based on panel methods, that is, following the sample of people across time and examining whether our results hold or change. This will also allow better identification of an eventual moderating effect of the pandemic on the relationship between employment trajectories and health.

Our employment trajectories could also be subject to recall bias or measurement error, as these were reconstructed retrospectively using life history calendars. However, these data collection methods have been shown to be reliable when applied by trained interviewers [42].

Finally, while we analyzed a widely validated scale of mental health, other traditional scales not included in the survey used in this research, such as the Center for Epidemiological Studies-Depression (CES-D), the Geriatric Depression Scale (GDS), or overall indicators of loneliness, life satisfaction, and subjective wellbeing, could be examined in order to strengthen our findings.

## 5. Conclusions

Relying both on a life course perspective and on rich longitudinal quantitative data, this study explored how diverse employment trajectories across adulthood are related to older people’s mental health before and after the onset of the COVID-19 pandemic in Chile, a developing country in the 20th century with no research in this field. Our findings indicate that, even when controlling for traditional risk factors, formal and stable labor force patterns in adulthood are associated with the lowest burden of mental health problems in old age, both before and after the onset of the overwhelming pandemic that mankind has faced for the last two years. Our results show the importance of promoting formal employment policies in countries with institutional contexts such as those in Chile. In particular, stability and formality together act as a protective factor against disruptive events such as a global pandemic with unprecedented effects on people’s mental health globally.

This research contributes substantively to the field of life course studies focused, first, on later life mental health, second, on the potential influence of earlier experiences in various domains of life among a specific cohort, and third, on the relationship between these trajectories and wellbeing after the COVID-19 pandemic. The nationally-representative and longitudinal data used in this research will allow us in the future to continue understanding the diverse long-term results of the pandemic in the social, economic, and health domains.

## Figures and Tables

**Table 1:** Types of adulthood employment trajectories and proportion (%) across the study samples.

Employment Trajectory Type	Description	Proportion in Baseline Sample (N = 3782)	Proportion in Merged Sample T1 (N = 1487)	Proportion in Merged Sample T2 (N = 1252)
Type 1. Conventional work life cycle	Dependent employees working persistently under formal, full-time, and stable employment conditions, who contribute continuously to social security.	44.0	43.2	43.6
Type 2. Out of the labor force	Includes individuals who remain inactive, unemployed, or are looking for a job during the whole period of interest, and who consequently did not contribute to social security at all.	31.4	33.8	31.6
Type 3. Full-time self-employed not contributing	Includes self-employed workers who do not contribute to social security at all (as expected, as they were not obliged to) and who have always been self-employed or switched to self-employment after a brief stint as dependent employees (generally after age 35).	11.2	9.6	10.5
Type 4. Wage-earners not contributing	Comprises dependent employees who do not contribute to social security, among which about a third start contributing toward the end of their careers.	5.3	5.4	5.4
Type 5. Full-time self-employed contributing	Groups the full-time self-employed who contribute to social security from the beginning of their careers.	3.8	3.9	4.0
Type 6. Part-time self-employed not contributing	Includes part-time self-employed workers who do not contribute to social security, among which some move to full-time positions in the late period of their working life.	2.5	2.4	2.4
Type 7. Part-time wage-earners contributing	Includes part-time dependent employees who contribute to social security, among which a small group from age 50 onwards start to move to full-time jobs.	1.9	1.7	2.5

Note: Merged samples correspond to the result of merging the baseline data with waves T1 and T2.

**Table 2:** Descriptive statistics of mental health outcomes and gender across study samples.

	Before Pandemic Onset		After Pandemic Onset	
Variables	Wave T1	Merged Sample T1	Wave T2	Merged Sample T2
**Continuous PHQ-9 scale (mean, SD)**	4.2	4.5	5.9	6.1
**Five-level PHQ-9 scale (%)**				
None	64.9	63.1	51.1	51.3
Mild	21.3	21.6	25.2	23.2
Moderate	8.2	9.0	13.2	13.6
Moderately severe	3.6	3.9	6.8	8.0
Severe	2.1	2.4	3.7	3.9
**Two-level PHQ-9 scale (%)**				
Non-depressed	86.2	84.7	74.7	74.5
Depressed	13.8	15.3	25.3	25.5
**Gender (%)**				
Men	43.1	42.3	43.3	43.4
Women	56.9	57.7	56.7	56.6

Note: Merged samples correspond to the result of merging the baseline data with waves T1 and T2. Sample sizes: T1 = 1487, T2 = 1252. SD = Standard deviation.

## Data Availability

The data presented in this study are available on request from the first and corresponding author.

## Appendix A

**Table A1:** Descriptive statistics of control variables for the whole sample and for each employment trajectory type.

	Whole Sample		Type 1. Conventional Work Life Cycle		Type 2. Out of the Labor Force		Type 3. Full-Time Self-Employed Not Contributing		Type 4. Wage-Earners Not Contributing		Type 5. Full-Time Self-Employed Contributing		Type 6. Part-Time Self-Employed Not Contributing		Type 7. Part-Time Wage-Earners Contributing	
Variables	T1	T2	T1	T2	T1	T2	T1	T2	T1	T2	T1	T2	T1	T2	T1	T2
**Age (Mean, SD)**	67.7 (4.3)	67.4 (4.3)	67.8 (4.4)	67.7 (4.3)	67.2 (4.3)	66.8 (4.3)	67.7 (4.2)	67.5 (4.5)	67.7 (4.1)	66.8 (4.5)	68.1 (4.1)	68.5 (4.5)	68.3 (4.5)	68.1 (3.4)	69.3 (3.9)	68.3 (3.2)
**Gender (%)**																
Men	41.2	43.4	59.2	60.6	7.1	7.8	72.9	74.8	36.8	47.1	80.0	66.0	48.4	43.3	30.4	16.1
Women	58.8	56.6	40.8	39.4	92.9	92.2	27.1	25.2	63.2	52.9	20.0	34.0	51.6	56.7	69.6	83.9
**Education (%)**																
None or primary	43.2	40.1	34.2	28.9	51.7	51.3	49.2	48.9	52.9	58.8	32.7	30.0	64.5	53.3	34.8	22.6
Secondary	44.8	44.2	48.0	46.5	43.5	42.4	44.9	43.5	41.2	39.7	43.6	48.0	35.5	46.7	21.7	29.0
Tertiary	12.0	15.7	17.8	24.6	4.8	6.3	5.9	7.6	5.9	1.5	23.6	22.0	0.0	0.0	43.5	48.4
**Number of chronic diseases (Mean, SD)**	1.1 (1.1)	1.1 (1.1)	0.9 (1.0)	0.9 (1.0)	1.4 (1.2)	1.3 (1.2)	0.9 (1.1)	1.0 (1.2)	1.1 (1.1)	1.1 (1.1)	1.1 (1.2)	1.1 (1.2)	1.5 (1.5)	1.1 (1.4)	1.0 (1.1)	1.1 (1.1)
**Number of functional limitations** **(Mean, SD)**	0.4 (1.1)	0.3 (0.9)	0.3 (0.9)	0.3 (0.9)	0.5 (1.3)	0.4 (1.0)	0.4 (1.3)	0.3 (0.9)	0.3 (0.9)	0.3 (0.9)	0.3 (1.0)	0.2 (0.6)	0.3 (0.7)	0.3 (0.9)	0.6 (1.2)	0.4 (1.0)
**Number of children** **(Mean, SD)**	2.9 (1.7)	2.7 (1.7)	2.6 (1.6)	2.5 (1.5)	3.1 (1.7)	3.0 (1.8)	3.2 (2.2)	2.8 (1.7)	3.0 (1.5)	2.6 (1.7)	2.8 (1.8)	2.9 (1.8)	2.9 (1.6)	2.6 (1.5)	2.5 (1.8)	2.5 (1.9)
**Household income decile** **(Mean, SD)**	5.4 (2.8)	5.4 (2.8)	5.3 (2.9)	5.3 (2.9)	5.7 (2.7)	5.7 (2.7)	5.1 (2.7)	5.3 (2.8)	5.2 (2.4)	4.7 (2.7)	4.9 (3.2)	4.6 (3.0)	5.7 (2.9)	5.1 (2.8)	5.3 (3.1)	5.9 (2.8)
**Marital status (%)**																
Divorced	12.7	12.1	13.8	12.8	13.0	11.4	11.0	13.7	11.8	7.4	5.5	12.0	12.9	13.3	8.7	12.9
Partnered	69.2	67.4	68.9	67.0	67.5	67.7	72.0	67.2	66.2	70.6	85.5	74.0	74.2	73.3	60.9	48.4
Single	10.0	11.8	11.7	13.7	8.9	9.1	6.8	10.7	7.4	16.2	7.3	4.0	9.7	6.7	21.7	25.8
Widow	8.1	8.6	5.7	6.4	10.6	11.9	10.2	8.4	14.7	5.9	1.8	10.0	3.2	6.7	8.7	12.9
**Drink wine (%)**																
Yes	27.8	30.4	33.6	38.5	18.0	17.4	36.4	33.6	27.9	36.8	38.2	34.0	19.4	33.3	26.1	19.4
No	72.2	69.6	66.4	61.5	82.0	82.6	63.6	66.4	72.1	63.2	61.8	66.0	80.6	66.7	73.9	80.6
**Drink liquor (%)**																
Yes	7.7	8.2	9.7	10.8	3.5	4.3	14.4	9.2	11.8	11.8	5.5	6.0	0.0	6.7	13.0	6.5
No	92.3	91.8	90.3	89.2	96.5	95.7	85.6	90.8	88.2	88.2	94.5	94.0	100	93.3	87.0	93.5
**Smoke (%)**																
Yes	21.9	23.2	22.1	24.1	21.6	22.3	25.4	26.7	22.1	26.5	21.8	18.0	19.4	16.7	8.7	12.9
No	78.1	76.8	77.9	75.9	78.4	77.7	74.6	73.3	77.9	73.5	78.2	82.0	80.6	83.3	91.3	87.1
**Drink beer (%)**																
Yes	18.1	21.3	24.5	27.9	8.9	10.1	26.3	26.7	19.1	23.5	23.6	30.0	3.2	20.0	8.7	6.5
No	81.9	78.7	75.5	72.1	91.1	89.9	73.7	73.3	80.9	76.5	76.4	70.0	96.8	80.0	91.3	93.5

Note: ‘T1′ and ‘T2′ correspond to the result of the merging of the baseline data with waves T1 and T2, respectively.

## Appendix B

**Table A2:** Bivariate associations between employment trajectories and mental health outcomes before and after the onset of the COVID-19 pandemic.

	Formal Employment Trajectories						Informal Employment Trajectories						Non-Employment Trajectories	
	Type 1. Conventional Work Life Cycle		Type 5. Full-Time Self-Employed Contributing		Type 7. Part-Time Wage-Earners Contributing		Type 3. Full-Time Self-Employed Not Contributing		Type 4. Wage-Earners Not Contributing		Type 6. Part-Time Self-Employed Not Contributing		Type 2. Out of the Labor Force	
**Variables**	**T1**	**T2**	**T1**	**T2**	**T1**	**T2**	**T1**	**T2**	**T1**	**T2**	**T1**	**T2**	**T1**	**T2**
**Continuous PHQ-9 scale** **(mean, SD)**	3.5 (4.9)	5.2 (5.8)	4.9 (6.3)	6.5 (5.9)	4.2 (5.7)	5.6 (6.2)	4.8 (5.9)	6.6 (6.1)	4.8 (5.6)	6.7 (6.8)	4.7 (5)	6.6 (5.5)	5.6 (5.6)	7.1 (6.3)
**Five-level PHQ-9 scale (%)**														
None	70.3	58.2	63.9	48.1	67.8	52.3	65.2	48.7	62.3	48.6	57.6	39.5	52.6	43.6
Mild	19.1	20.3	21.2	26.3	19.7	28.7	18.2	23.7	19.1	24.8	24.9	30.1	26.1	24.9
Moderate	6.3	12.1	3.1	11.7	4.2	13	8.8	14.3	11.2	13.2	8.1	22.8	13.1	15.1
Moderately severe	3.1	6.2	8.9	12.0	4.1	0.4	3.9	8.1	5.6	6.2	8.1	3.3	4.1	11.2
Severe	2.2	3.2	2.9	1.9	4.2	5.6	3.9	5.2	1.8	7.2	1.3	3.3	3.1	5.2
**Two-level PHQ-9 scale (%)**														
Depressed	1.00	1.00	1.39 (0.53)	1.23 (0.36)	1.25 (0.74)	.92 (0.38)	1.46 (0.39)	1.26 (0.24)	1.83 (0.56)	1.25 (0.32)	1.85 (0.79)	1.42 (0.49)	1.93 (0.56)	1.47 (0.19)

Note SD = Standard deviation. We assessed the bivariate relationship between employment trajectory types and the continuous and five-level scale using ANOVA and chi-square tests, respectively. For the two-level PHQ-9, we used a Poisson regression model with the trajectory types as an independent variable to estimate prevalence ratios, with Type 1 as a reference category. The ANOVA test is statistically significant at a *p*-value < 0.05. The association with the five-level PHQ-9 scale is statistically significant at a *p*-value < 0.1. ‘T1′ and ‘T2′ correspond to the result of the merging of the baseline data with waves T1 and T2, respectively. Sample sizes: T1 = 1487, T2 = 1252.

## Appendix C

**Table A3:** Multivariate linear regression models on continuous PHQ-9 scale before and after the onset of the COVID-19 pandemic (collapsed trajectory types).

	Before Pandemic Onset	After Pandemic Onset
	T1	T2
Variables	Continuous PHQ-9 Scale	Continuous PHQ-9 Scale
**Employment trajectory type**		
Formal employment trajectories (ref)	-	-
Informal employment trajectories	0.79 (0.41)	1.04 * (0.51)
Non-employment trajectories	0.18 (0.38)	−0.09 (0.49)
Constant	8.96 *** (2.39)	8.39 ** (3.078)
R-Squared	0.12	0.11
N	1315	1094

Note: ß coefficients reported. Standard errors are in parenthesis. Regression models were adjusted by the following variables that typically affect mental health: marital status, education level, number of children, household income decile, age, gender, tobacco use, beer consumption, wine consumption, liquor consumption, number of functional limitations, and number of chronic conditions. *p* values: * = *p* < 0.05, ** = *p* < 0.01, *** = *p* < 0.001. Statistically significant results are highlighted in grey. ‘T1′ and ‘T2′ correspond to the result of the merging of the baseline data with waves T1 and T2, respectively.

## Appendix D

**Table A4:** Multivariate linear regression models at two time points (all trajectory types).

	Before Pandemic Onset	After Pandemic Onset
	T1	T2
Variables	Continuous PHQ-9 Scale	Continuous PHQ-9 Scale
**Employment trajectory type** (Ref: Type 1. Conventional work life cycle)	-	-
*Formal employment trajectories*		
Type 5. Full-time self-employed contributing	1.68 * (0.73)	1.07 (0.99)
Type 7. Part-time wage-earners contributing	0.01 (1.1)	−0.41 (1.15)
*Informal employment trajectories*		
Type 3. Full-time self-employed not contributing	1.13 * (0.524)	1.59 * (0.628)
Type 4. Wage-earners not contributing	0.67 (0.67)	0.27 (0.84)
Type 6. Part-time self-employed not contributing	0.75 (0.96)	0.71 (1.3)
*Non-employment trajectories*		
Type 2. Out of the labor force	0.26 (0.39)	−0.12 (0.51)
Constant	8.90 *** (2.39)	8.48 ** (3.16)
R-Squared	0.12	0.11
N	1315	1094

Note: ß coefficients reported. Standard errors are in parenthesis. Regression models adjusted by the following that typically affect mental health: marital status, education level, number of children, household income decile, age, gender, tobacco use, beer consumption, wine consumption, liquor consumption, number of functional limitations, and number of chronic conditions. *p* values: * = *p* < 0.05, ** = *p* < 0.01, *** = *p* < 0.001. Statistically significant results are highlighted in grey. ‘T1′ and ‘T2′ correspond to the result of the merging of the baseline data with waves T1 and T2, respectively.

## References

[1] Villalobos Dintrans P., Browne J., Madero-Cabib I. (2021). It Is Not Just Mortality: A Call From Chile for Comprehensive COVID-19 Policy Responses among Older People. J. Gerontol. Ser. B.

[2] Girgus J.S., Yang K. (2015). Gender and Depression. Curr. Opin. Psychol..

[3] Horesh D., Lev-Ari R.K., Hasson-Ohayon I. (2020). Risk Factors for Psychological Distress during the COVID-19 Pandemic in Israel: Loneliness, Age, Gender, and Health Status Play an Important Role. Br. J. Health Psychol..

[4] Gustavsson J., Beckman L. (2020). Compliance to Recommendations and Mental Health Consequences among Elderly in Sweden during the Initial Phase of the COVID-19 Pandemic—A Cross Sectional Online Survey. Int. J. Environ. Res. Public Health.

[5] Arpino B., Pasqualini M., Bordone V., Solé-Auró A. (2021). Older People’s Nonphysical Contacts and Depression during the COVID-19 Lockdown. Gerontologist.

[6] Fingerman K.L., Ng Y.T., Zhang S., Britt K., Colera G., Birditt K.S., Charles S.T. (2021). Living Alone during COVID-19: Social Contact and Emotional Well-Being among Older Adults. J. Gerontol. Ser. B.

[7] Bergman Y.S., Cohen-Fridel S., Shrira A., Bodner E., Palgi Y. (2020). COVID-19 Health Worries and Anxiety Symptoms among Older Adults: The Moderating Role of Ageism. Int. Psychogeriatr..

[8] García-Fernández L., Romero-Ferreiro V., López-Roldán P.D., Padilla S., Rodriguez-Jimenez R. (2020). Mental Health in Elderly Spanish People in Times of COVID-19 Outbreak. Am. J. Geriatr. Psychiatry.

[9] Bui C.N., Peng C., Mutchler J.E., Burr J.A. (2021). Race and Ethnic Group Disparities in Emotional Distress among Older Adults During the COVID-19 Pandemic. Gerontologist.

[10] Polenick C.A., Perbix E.A., Salwi S.M., Maust D.T., Birditt K.S., Brooks J.M. (2021). Loneliness during the COVID-19 Pandemic Among Older Adults with Chronic Conditions. J. Appl. Gerontol..

[11] Pan H., Fokkema T., Switsers L., Dury S., Hoens S., De Donder L. (2021). Older Chinese Migrants in Coronavirus Pandemic: Exploring Risk and Protective Factors to Increased Loneliness. Eur. J. Ageing.

[12] Wu Q., Xu Y., Jedwab M. (2021). Custodial Grandparent’s Job Loss during the COVID-19 Pandemic and Its Relationship with Parenting Stress and Mental Health. J. Appl. Gerontol..

[13] Whitehead B.R. (2021). COVID-19 as a Stressor: Pandemic Expectations, Perceived Stress, and Negative Affect in Older Adults. J. Gerontol. Ser. B.

[14] Creese B., Khan Z., Henley W., O’Dwyer S., Corbett A., Vasconcelos Da Silva M., Mills K., Wright N., Testad I., Aarsland D. (2021). Loneliness, Physical Activity, and Mental Health during COVID-19: A Longitudinal Analysis of Depression and Anxiety in Adults over the Age of 50 between 2015 and 2020. Int. Psychogeriatr..

[15] Pierce M., Hope H., Ford T., Hatch S., Hotopf M., John A., Kontopantelis E., Webb R., Wessely S., McManus S. (2020). Mental Health before and during the COVID-19 Pandemic: A Longitudinal Probability Sample Survey of the UK Population. Lancet Psychiatry.

[16] Chaaya M., Sibai A.M., Tabbal N., Chemaitelly H., El Roueiheb Z., Slim Z.N. (2010). Work and Mental Health: The Case of Older Men Living in Underprivileged Communities in Lebanon. Ageing Soc..

[17] Min J., Ailshire J., Crimmins E.M. (2016). Social Engagement and Depressive Symptoms: Do Baseline Depression Status and Type of Social Activities Make a Difference?. Age Ageing.

[18] Worach-Kardas H., Kostrzewski S. (2014). Quality of Life and Health State of Long—Term Unemployed in Older Production Age. Appl. Res. Qual. Life.

[19] Nemoto Y., Takahashi T., Nonaka K., Hasebe M., Koike T., Minami U., Murayama H., Matsunaga H., Kobayashi E., Fujiwara Y. (2020). Working for Only Financial Reasons Attenuates the Health Effects of Working beyond Retirement Age: A 2-year Longitudinal Study. Geriatr. Gerontol. Int..

[20] Behncke S. (2012). Does Retirement Trigger Ill Health?. Health Econ..

[21] Dave D., Rashad I., Spasojevic J. (2008). The Effects of Retirement on Physical and Mental Health Outcomes. South. Econ. J..

[22] Gallo W.T., Bradley E.H., Dubin J.A., Jones R.N., Falba T.A., Teng H.-M., Kasl S.V. (2006). The Persistence of Depressive Symptoms in Older Workers Who Experience Involuntary Job Loss: Results From the Health and Retirement Survey. J. Gerontol. Ser. B Psychol. Sci. Soc. Sci..

[23] Tokuda Y., Ohde S., Takahashi O., Shakudo M., Yanai H., Shimbo T., Fukuhara S., Hinohara S., Fukui T. (2008). Relationships between Working Status and Health or Health-Care Utilization among Japanese Elderly. Geriatr. Gerontol. Int..

[24] Engels M., Wahrendorf M., Dragano N., McMunn A., Deindl C. (2021). Multiple Social Roles in Early Adulthood and Later Mental Health in Different Labour Market Contexts. Adv. Life Course Res..

[25] Hoven H., Wahrendorf M., Goldberg M., Zins M., Siegrist J. (2020). Cumulative Disadvantage during Employment Careers—The Link between Employment Histories and Stressful Working Conditions. Adv. Life Course Res..

[26] Settersten R.A., Bernardi L., Härkönen J., Antonucci T.C., Dykstra P.A., Heckhausen J., Kuh D., Mayer K.U., Moen P., Mortimer J.T. (2020). Understanding the Effects of COVID-19 through a Life Course Lens. Adv. Life Course Res..

[27] Wahrendorf M., Blane D., Bartley M., Dragano N., Siegrist J. (2013). Working Conditions in Mid-Life and Mental Health in Older Ages. Adv. Life Course Res..

[28] Madero-Cabib I., Biehl A., Sehnbruch K., Calvo E., Bertranou F. (2019). Private Pension Systems Built on Precarious Foundations: A Cohort Study of Labor-Force Trajectories in Chile. Res. Aging.

[29] Madero-Cabib I., Azar A., Guerra J. (2022). Simultaneous Employment and Depressive Symptom Trajectories around Retirement Age in Chile. Aging Ment. Health.

[30] Engels M., Weyers S., Moebus S., Jöckel K.-H., Erbel R., Pesch B., Behrens T., Dragano N., Wahrendorf M. (2019). Gendered Work-Family Trajectories and Depression at Older Age. Aging Ment. Health.

[31] Giudici F., Morselli D. (2019). 20 Years in the World of Work: A Study of (Nonstandard) Occupational Trajectories and Health. Soc. Sci. Med..

[32] Majeed T., Forder P.M., Mishra G., Kendig H., Byles J.E. (2017). Exploring Workforce Participation Patterns and Chronic Diseases Among Middle-Aged Australian Men and Women Over the Life Course. J. Aging Health.

[33] Zella S., Harper S. (2020). The Impact of Life Course Employment and Domestic Duties on the Well-Being of Retired Women and the Social Protection Systems That Frame This. J. Aging Health.

[34] Wickrama K.A.S., King V.A., O’Neal C.W., Lorenz F.O. (2019). Stressful Work Trajectories and Depressive Symptoms in Middle-Aged Couples: Moderating Effect of Marital Warmth. J. Aging Health.

[35] Wahrendorf M., Hoven H., Deindl C., Lunau T., Zaninotto P. (2021). Adverse Employment Histories, Later Health Functioning and National Labor Market Policies: European Findings Based on Life-History Data From SHARE and ELSA. J. Gerontol. Ser. B.

[36] Madero-Cabib I., Biehl A. (2021). Lifetime Employment–Coresidential Trajectories and Extended Working Life in Chile. J. Econ. Ageing.

[37] Baader T., Molina J.L., Venezian S., Rojas C., Farías R., Fierro-Freixenet C., Backenstrass M., Mundt C. (2012). Validación y utilidad de la encuesta PHQ-9 (Patient Health Questionnaire) en el diagnóstico de depresión en pacientes usuarios de atención primaria en Chile. Rev. Chil. Neuro-Psiquiatr..

[38] Kroenke K., Spitzer R.L., Williams J.B.W. (2001). The PHQ-9: Validity of a Brief Depression Severity Measure. J. Gen. Intern. Med..

[39] R Core Team (2019). A Language and Environment for Statistical Computing.

[40] Höppner J. (2021). How Does Self-employment Affect Pension Income? A Comparative Analysis of European Welfare States. Soc. Policy Adm..

[41] Lewis K., Walker E.A. (2011). Self-Employment: Policy Panacea for an Ageing Population?. Small Enterp. Res..

[42] Morselli D., Le Goff J.M., Gauthier J.A. (2019). Self-administered event history calendars: A possibility for surveys?. Contemp. Soc. Sci..

